# Prospective data collection and analysis of perforations and tears of latex surgical gloves during primary endoprosthetic surgeries

**DOI:** 10.3205/dgkh000285

**Published:** 2016-12-20

**Authors:** Sarah Zaatreh, Andreas Enz, Annett Klinder, Tony König, Lena Mittelmeier, Günther Kundt, Wolfram Mittelmeier

**Affiliations:** 1 Department of Orthopedics, University Medicine Rostock, Rostock, Germany; 2 Institutes for Biostatistics and Informatics in Medicine and Ageing Research, University of Rostock, Rostock, Germany

**Keywords:** surgical gloves, latex, perforations, tears, orthopedic surgery, ISO DIN 455-1, joint replacement, infection risk

## Abstract

**Introduction:** Surgical gloves are used to prevent contamination of the patient and the hospital staff with pathogens. The aim of this study was to examine the actual effectiveness of gloves by examining the damage (perforations, tears) to latex gloves during surgery in the case of primary hip and knee prosthesis implantation.

**Materials and methods:** Latex surgical gloves used by surgeons for primary hip and knee replacement surgeries were collected directly after the surgery and tested using the watertightness test according to ISO EN 455-1:2000.

**Results:** 540 gloves were collected from 104 surgeries. In 32.7% of surgeries at least one glove was damaged. Of all the gloves collected, 10.9% were damaged, mainly on the index finger. The size of the perforations ranged from ≤1 mm to over 5 mm. The surgeon’s glove size was the only factor that significantly influenced the occurrence of glove damage. Surgeon training level, procedure duration, and the use of bone cement had no significant influence.

**Conclusions:** Our results highlight the high failure rate of surgical gloves. This has acute implications for glove production, surgical practice, and hygiene guidelines. Further studies are needed to detect the surgical steps, surface structures, and instruments that pose an increased risk for glove damage.

## Zusammenfassung

**Einleitung:** OP-Handschuhe werden verwendet, um Kontaminationen des Patienten und des Krankenhauspersonals mit Krankheitserregern zu verhindern. Das Ziel dieser Studie war es, die tatsächliche Wirksamkeit der OP-Handschuhe durch Untersuchung der Beschädigungen (Perforationen, Risse), die während der Operation im Fall von Hüft- und Knieendoprothesen-Implantation entstehen können, zu untersuchen.

**Material und Methoden:** Latex-OP-Handschuhe, die Chirurgen bei dem Einbau einer primären Hüft- und Knieendoprothese verwendeten, wurden direkt nach der Operation gesammelt und auf Wasserdichtigkeit nach der ISO EN 455-1:2000 getestet.

**Ergebnisse:** 540 Handschuhe aus 104 Operationen wurden gesammelt und untersucht. In 32,7% der Operationen wurde zumindest ein Handschuh beschädigt. Von allen gesammelten Handschuhen waren 10,9% beschädigt, vor allem am Zeigefinger. Die Größe der Perforationen lag im Bereich von unter 1 mm bis mehr als 5 mm. Die Handschuhgröße des Operateurs war der einzige signifikante Einflussfaktor für das Auftreten von Handschuhschäden. Das chirurgische Ausbildungsniveau, die Dauer des Eingriffs und die Verwendung von Knochenzement zeigten keinen signifikanten Einfluss.

**Schlussfolgerung:** Unsere Ergebnisse zeigen, dass die derzeit verwendeten OP-Handschuhe in der Tat eine hohe Ausfallrate aufweisen. Dies hat akute Auswirkungen auf die Handschuhproduktion, chirurgische Praxis und Hygienerichtlinien. Weitere Studien sind erforderlich, um die einzelnen Operationshandgriffe, verschiedene Oberflächenstrukturen und Instrumente zu erkennen, die ein erhöhtes Risiko für Handschuh-Schäden darstellen.

## Background

Intact surgical gloves function as an important barrier against the transmission of bacteria between the surgical team and their patient [1], [2], [3], [4]. Cross contaminations and surgical site infections are a major risk in orthopedic surgery [5], [6], [7], [8], [9], [10]. Although there are now strict guidelines regarding the prevention of such complications, the number of infections is still high. Besides perioperative application of antibiotics and sterile conditions, the use of surgical gloves is supposed to prevent contamination of the patient and the hospital staff [11], [12], [13]. Yet trials have confirmed that perforations and tears of gloves are common [4], [5], [8], [9].

However, there is no standardized changing system specified for gloves during operations. The ISO EN 455-1:2000 [14] merely requires impermeableness and tear resistance as a prerequisite for gloves. However, the highly repetitive pressure and shear strain of the surgical routine are not reflected. Likewise, punctures resulting from sharp bone surfaces of implants, instruments, or cement surfaces are not taken into consideration [5].

The aim of this study was to evaluate the damage to latex gloves, which occurs during the implantation of prostheses in surgical orthopedics. The analysis followed the watertightness test according to ISO EN 455-1:2000. We compared the quantity and quality of glove damage in primary total knee and hip replacements.

## Materials and methods

### Study design and data collection

From May 1^st^, 2015 to May 1^st^, 2016 surgical gloves used during primary hip (ICD-9-CM code 81.51) and knee (ICD-9-CM code 81.54) replacement surgeries were collected at the Department of Orthopedics of the University Medicine Rostock. Throughout the study, 540 surgical gloves were retrieved from 104 elective primary endoprosthetic surgeries. Damage to surgical gloves was analyzed within 24 h after the surgery. The duration of the surgery, type of surgery (knee or hip), type of surgeon, number of used gloves, and glove size were recorded.

In the month after the collection period it was considered necessary to relate glove damage to some of the patient data. Therefore, we applied for ethical approval to access the patient’s data from their medical files that were unrelated to the study as part of standard clinical procedure. After ethical approval was given by the Local Ethical Committee of Rostock, Germany (registration number: A2016-0112), patient-related data (BMI, age, previous operation, date and type of operation) were collected retrospectively. From the 15 orthopedic surgeons participating in the study 14 were right-handed and one was left-handed. Seven were main surgeons and eight were surgeons in training. All surgeons expressed their consent to the examination of their gloves. 

### Surgical gloves

The standard gloves used during endoprosthetic surgery were sterile, powder-free disposable latex gloves for single use (Protexis^TM^, Cardinal Health, Dublin, Ohio, USA). The size of the gloves is the hand circumference across the palm in inches. The surgical procedure at the Orthopedics Department of University Medicine Rostock involves the use of double gloves, one pair worn over the other. We refer to the pair worn over the first pair as the outer gloves.

Intraoperatively, exchanges of gloves were carried out when damage to the gloves was noticed during surgery. The changing process involved removing the glove and then turning it inside out. 

### Collection and integrity testing of the gloves 

After an operation, all gloves (outer and inner) were safely kept. The samples were collected in a plastic bag, sealed, and labeled. The name of the patient, the patient’s date of birth, the name of the surgeon, and the type and date of the operation were recorded.

**Figure 1:**
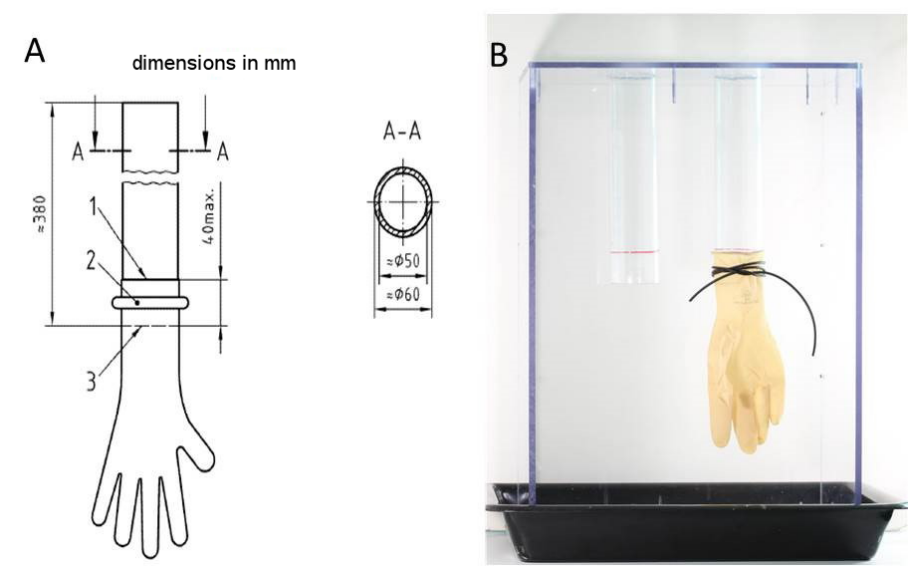
Watertightness tube apparatus to find perforations and tears via freedom from holes testing for used medical surgical gloves. A) Technical drawing of the watertightness tube / filling tube according to ISO EN 455-1:2000 [14]. B) Manufactured watertightness tube made out of polycarbonate with two cylinders of six centimeters outer diameter; glove was stretched over each of the cylinders up to a maximum of four centimeters and attached with a rubber seal.

The investigation of micro perforations and tears of the surgical gloves was performed within 24 h by using the freedom from holes testing method described in the ISO EN 455-1:2000, Medical gloves for single use Part 1: Requirements and testing for freedom from holes, watertightness test [14]. A specifically manufactured watertightness measuring apparatus was made out of polycarbonate with two cylinders that have an outer diameter of 6 cm according to the technical drawing of ISO EN 455-1:2000. This apparatus was used to inspect the gloves for holes (Figure 1 (Fig. 1)). One glove was stretched over each of the cylinders up to a maximum of 4 cm and attached with a rubber seal to avoid slipping. Blood residues were removed carefully. By pulling each finger of the glove, the detection of even small damages was ensured. Thereafter, the gloves were filled with 1,000 ± 50 ml warm water (room temperature: 15–25°C). The stretched gloves were tested immediately and after an additional 3 min. Perforations or tears were confirmed when a drop of water or a jet leaked from the glove.

The dimensions of the perforations and tears were measured afterwards with a plastic goniometer (Kirchner & Wilhelm GmbH & Co. KG, Asperg, Germany) and the location of the damage was classified according to the finger on which it occurred. Overview images of the damages via 3D laser scanning microscopy (VK-X260, Keyence Corporation, Osaka, Japan) were also taken.

### Statistical analysis

All data were stored and analyzed using the SPSS statistical package version 22 (IBM Corp., New York, USA). Descriptive statistics were computed for continuous and categorical variables. The statistics computed included means and standard deviations (SD) of continuous variables and are shown as mean ± SD or as frequencies [n] with percentages in brackets for categorical factors. For categorical factors, comparisons between patients with and without glove damage were performed by Fischer’s exact test (two categories) or by Pearson’s chi-squared test (more than two categories). Testing for differences of continuous variables between study groups created by existence of glove damage was accomplished by the two-sample t-test for independent samples or by the Mann-Whitney U-test by ranks as appropriate. Test selection was based on evaluating the variables for normal distribution, employing the Kolmogorov-Smirnov test. All p-values resulted from two-sided statistical tests and p≤0.05 was considered to be significant.

## Results

### General patient data and surgical procedure

A total of 540 surgical gloves were collected from the surgeries of 104 patients who participated in this study. Demographic data of the operated patients and the specific information about the surgical procedure are listed in Table 1 (Tab. 1). The majority of gloves were collected from total hip replacement operations (74.1% total hip versus 25.9% total knee replacements) based on the given ratio in the study center. Bone cement was used in the majority of analyzed surgeries. In about one third of operations at least one damaged glove was identified, equaling 10.9% of all collected gloves. 

### Association between patient data, operation specific data, and damage to surgical gloves

The factors analyzed for their influence on the occurrence of at least one damaged glove, are given in Table 2 (Tab. 2). Mann-Whitney U-test by ranks, t-test for independent samples and Fischer’s exact test were employed to test for significance. The surgeon’s glove size was the only factor with a significant influence on the occurrence of at least one damaged glove (p=0.031). When glove damage occurred, the average glove size was approximately 7.8 compared to 8.0 where no damage occurred. The glove size is the circumference across the palm of the hand in inches.

The data for the type of surgery (hip or knee), procedure duration, surgeon experience level (main or in training), use of bone cement showed no significant influence.

There was no association between the occurrence of damaged gloves and any of the patient data (gender, age, or BMI). 

### Position and dimension of the damage in surgical gloves

The highest incidence of glove damage occurred on the index finger at 62.7% (37/59) of all damaged gloves followed by the thumb, middle finger, and palm of the hand at 18.6% (11/59), 13.6% (8/59), and 5.1% (3/59) of all damaged gloves, respectively. No damage occurred on the ring finger or the little finger. Gloves from the dominant hand were more often affected by damage than gloves from the non-dominant hand (61.0% vs. 39.0%). When analyzing individual perforations, the incidence of damage was highest on the index finger of the dominant hand (39.0%) followed by the index finger of the non-dominant hand (23.7%), the thumb of the dominant hand (11.9%), and the middle finger of the dominant hand (8.5%). There was higher damage on the non-dominant hand (3.4%) compared to the dominant hand (1.7%) on the palm only. However, there was no significant difference in the distribution of the position of damage between the gloves on the dominant and non-dominant hands (p=0.795) (Table 3 (Tab. 3)).

The dimensions of the damage ranged from ≤1 mm up to more than 5 mm. Most tears had a macroscopic size of 2 mm and 3 mm, however, microscopic tears smaller than 1 mm did also occur (Table 4 (Tab. 4)).

**Figure 2:**
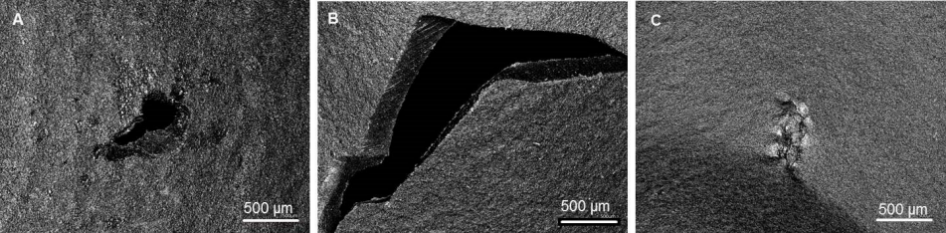
3D laser scanning microscopy images displaying different damage types of the surgical gloves A) 0.5 mm perforation at the right thumb. B) 3 mm tear at the right forefinger. C) 0.5 mm rubdown at the right middle finger. Images were taken at 20x magnification.

In order to characterize the type of damage to the gloves, 3D laser scanning microscopy was employed (Figure 2 (Fig. 2)). The types of damage we could identify were perforations, tears, and rubdown. In further studies, these images could be used to categorize the damage and correlate damage types to specific surgical steps. 

## Discussion

With damage in surgical gloves occurring in more than 30% of the total joint replacement surgeries studied, there are a considerable proportion of surgeries in which the barrier function attributed to gloves is potentially compromised. The number of perforations and tears demonstrates the heavy strain on gloves in orthopedic surgery. In our study, we did not distinguish inner and outer gloves due to the fact that the benefits of double gloving have been well established and the required effort did not promise to add sufficient additional value [3], [4], [15], [16], [17].

Regarding the factor of surgery duration, the average duration of the procedures recorded in our study was already lower than the 90 minutes, which is recommended as the time after which gloves should be changed [18]. An average operation time of less than 90 minutes might also be the reason why in contrast to other studies, which report an increase in the perforation rate with the duration of glove wear [15], [18], we were unable to demonstrate a significant association between the occurrence of damage and the operation time. 

The data on the location of the damage revealed that it was mainly the index finger that was affected. Contrary to the literature, in which the index finger of the non-dominant hand showed the highest perforation rate [15], [17], [18], we found that most of the damage occurred on the dominant hand. Further studies and analyses are needed to identify the specific steps, techniques, and instruments in surgery associated with an increased risk for glove damage. Chan et al. [19] found that damage to gloves in orthopedic procedures often occurred during the nailing procedure or internal fixings without wires and suggested that shear stress was responsible. Carter et al. [17], however, reported that most of the gloves were perforated in the period when the final components were implanted.

As demonstrated, 3D laser scanning microscopy can help to characterize and categorize different types of damage. This would allow further studies to link the type of damage to surgical procedures in order to identify causes of damage and work towards improving procedures and glove design. First developments in glove design were undertaken to prevent cross contamination even when the physical barrier is compromised by coating the inner lining of the glove with antimicrobial substances [20], [21]. However, the failure rates and observed damage highlight the importance of changing gloves as an immediate safety strategy and further studies are needed to estimate the efficiency of this strategy.

## Conclusions

Surgical gloves are universally used to prevent contamination of patient and hospital staff with pathogens. Their reliability is of highest importance, but is not guaranteed. We believe that the incidence of damaged gloves in 30% of surgeries and the distinct locality of breaches necessitate an extension of the relevant ISO standards to include realistic strain tests. Specifically for the types of surgeries in this study, the assumed risk factors are: handling of hard and sharp bone, specific surgical instruments, surgical steps requiring increased use of force and risk of slipping. Our findings, in accordance with existing studies on surgical gloves, suggest a need for improvement of surgical procedures, glove handling guidelines, and glove design. Further studies are needed to detect the surgical steps and instruments that pose an increased risk for glove damage. Furthermore, guidelines for replacing gloves during surgery concerning exposure to patients’ bodily liquids and mechanically demanding steps of surgery should be modified with regard to national hygiene recommendations.

## Notes

Trial identification number: A 2016-0112

### Competing interests

The authors declare that they have no competing interests.

### Authors’ contributions

All authors developed the study and performed analysis of the data. SZ, AK and LM drafted the manuscript; all authors revised and approved the final version of the manuscript.

### Acknowledgements

The authors want to thank the technical assistants Mr. Mario Jackzis and Ms. Laura Lux (Department of Orthopedics, University Medicine Rostock) for manufacturing the measuring apparatus and for imaging. 

## Figures and Tables

**Table 1:**
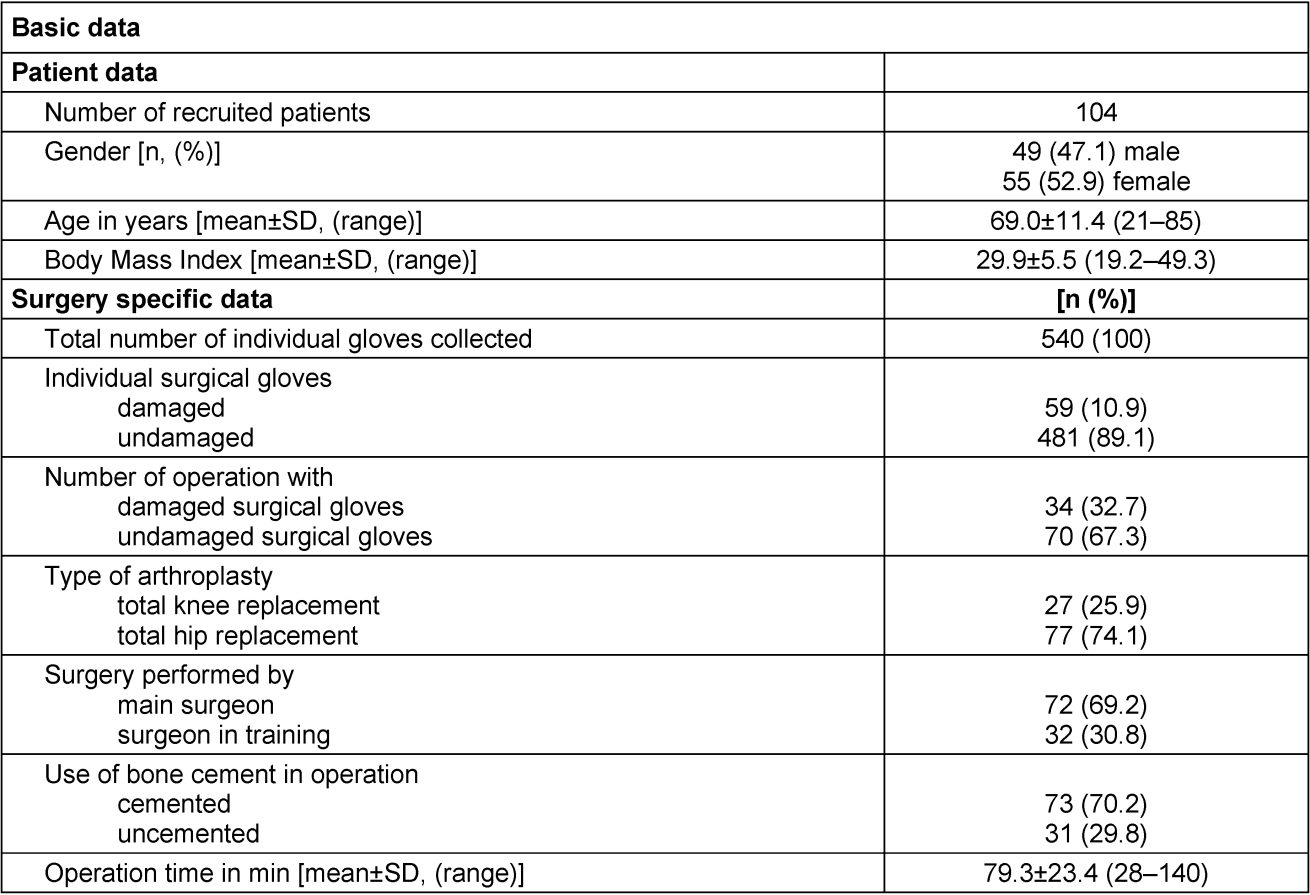
Overview of collected data

**Table 2:**
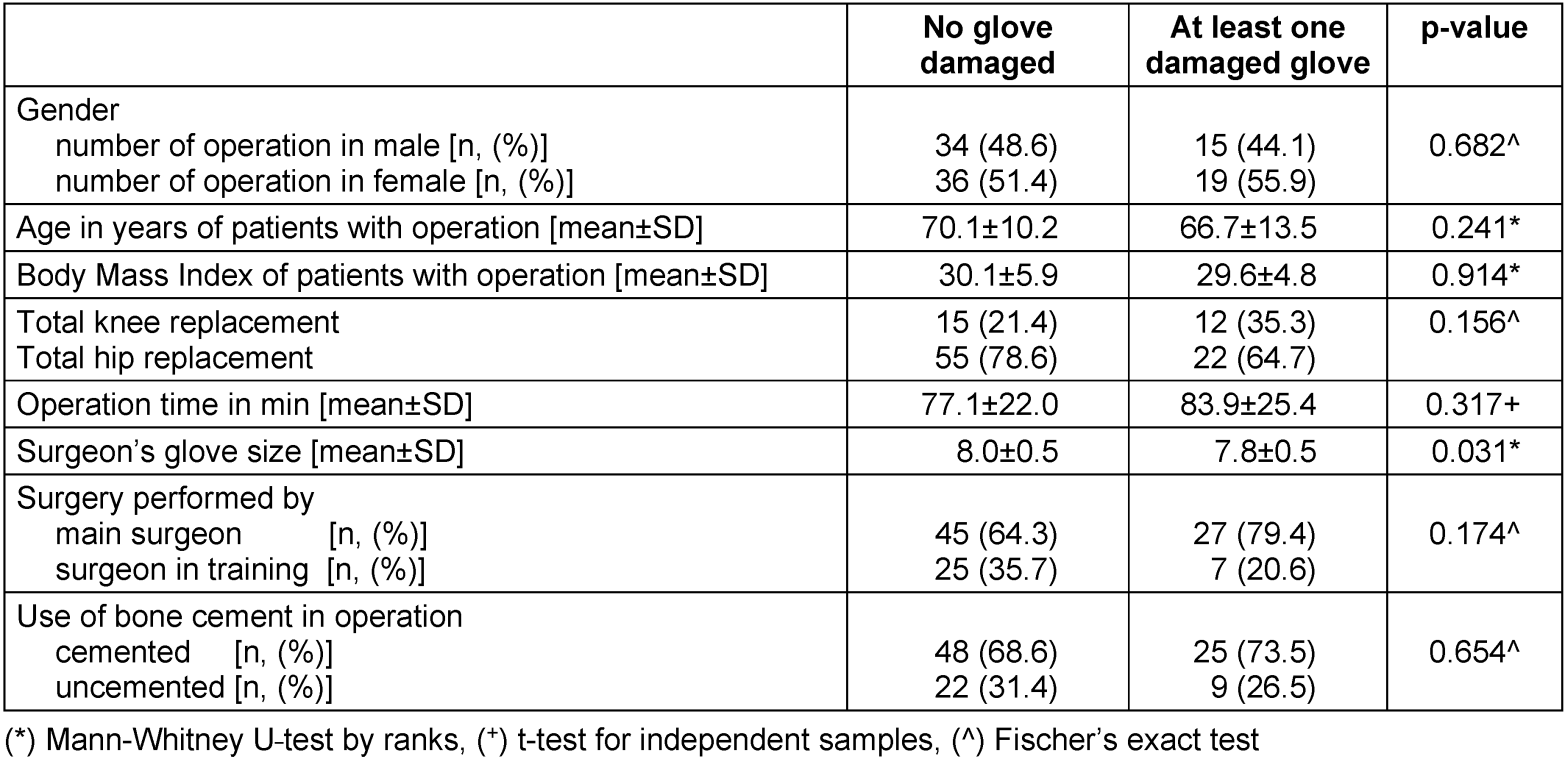
Statistical analysis of surgery data in relation to occurrence of glove damage

**Table 3:**
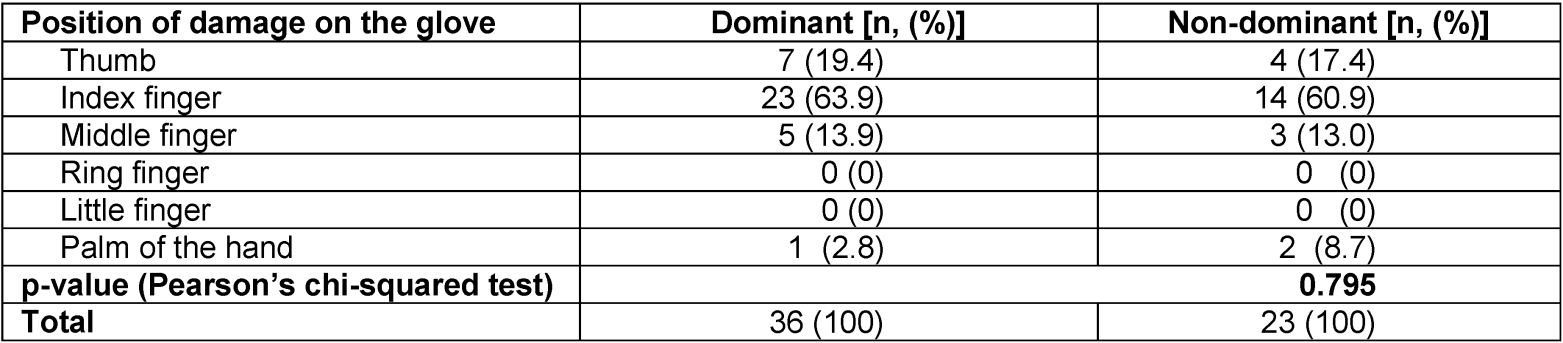
Comparison of position of glove damage between the dominant and the non-dominant hand

**Table 4:**
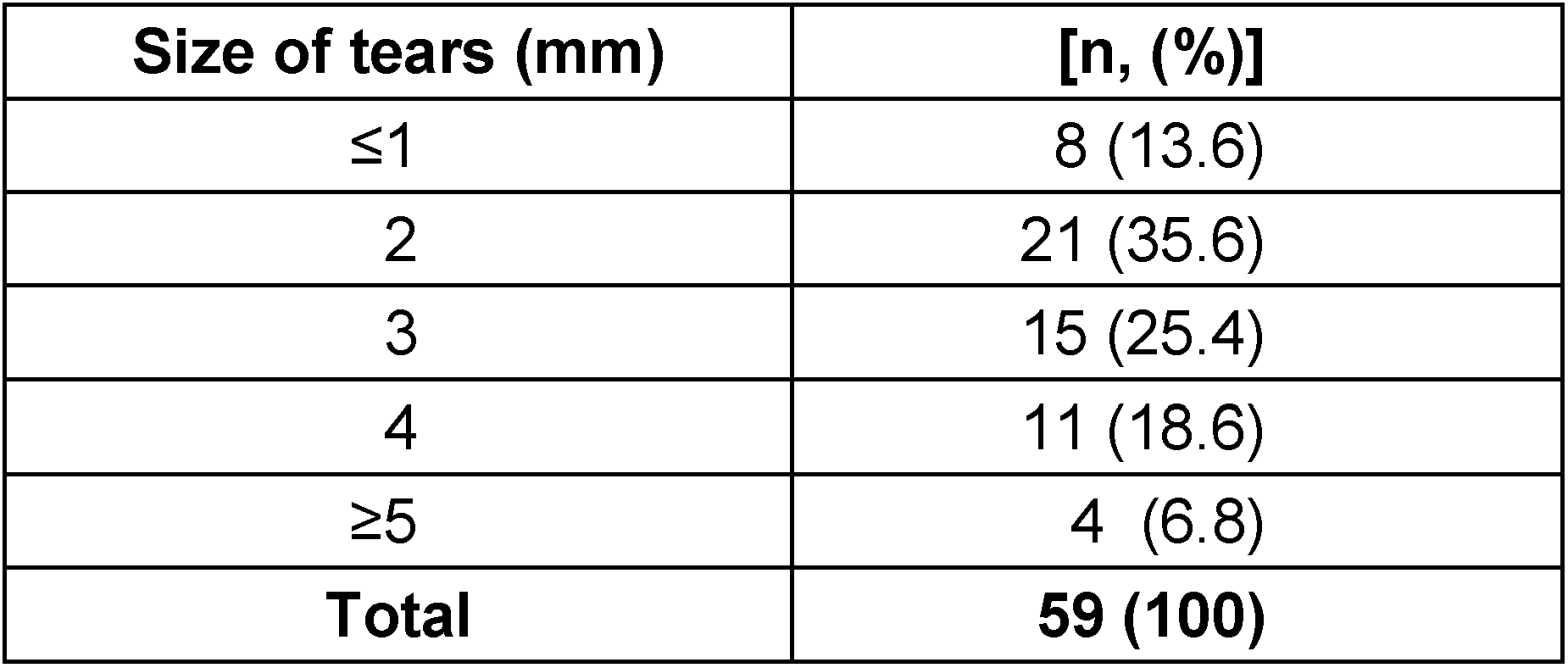
Size of tears at the gloves in mm
